# Quality Improvement Initiative for Assessing Allografts after Lung Transplantation

**DOI:** 10.1097/pq9.0000000000000146

**Published:** 2019-03-27

**Authors:** Don Hayes, Kerri L. Nicholson, Rebecca Miller, Ashley E. Nance, Stephen E. Kirkby, William Parker, Peter B. Baker

**Affiliations:** From the Departments of * Pediatrics; † Internal Medicine; ‡ Surgery, The Ohio State University College of Medicine, Columbus, Ohio.; § Section of Pulmonary Medicine, Nationwide Children’s Hospital, Columbus, Ohio.; ¶ Department of Anesthesiology and Pain Medicine, Nationwide Children’s Hospital, Columbus, Ohio.; ‖ Quality Improvement Services, Nationwide Children’s Hospital, Columbus, Ohio.; ** Department of Pathology, The Ohio State University College of Medicine, Columbus, Ohio.; †† Department of Pathology and Laboratory Medicine, Nationwide Children’s Hospital, Columbus, Ohio.

## Abstract

**Introduction:**

The histologic evaluation of lung allografts after transbronchial biopsy (TBBx) is a key component of the clinical care of lung transplant recipients. With established guidelines on diagnosing allograft rejection, no specific recommendations exist on timeliness to reaching a diagnosis and initiating therapy. A quality improvement initiative focused on 3 key stages of achieving a prompt diagnosis of acute cellular rejection including tissue processing, interpretation, and notification to the treating transplant pulmonologist was initiated to minimize time to treatment onset.

**Methods:**

We completed a single-center cohort study on all surveillance and clinically indicated TBBx from September 2006 to March 2018. The rapid tissue processing, interpretation, and notification system was instituted in March 2011 with data before this date serving as baseline.

**Results:**

We enrolled 28 patients who underwent 210 TBBx (1 excluded due to unknown notification date). Thirty-eight TBBx were included at baseline before implementation of the rapid tissue processing and communication system; 171 were included after implementation. Median time to notification following the change was 0 days (interquartile range, 0–1) compared with 1 day (interquartile range, 1–1) before the change (*P* < 0.001). After the change, same-day notification increased, with 110 (64%) TBBx resulting in same-day notification compared with 0 before (*P* < 0.001). We initiated treatment of acute cellular rejection on the day of diagnosis for the entire cohort.

**Conclusions:**

This quality improvement initiative resulted in more efficient analysis of TBBx of allografts in lung transplant recipients and faster communication of results to the clinical team.

## INTRODUCTION

Flexible fiberoptic bronchoscopy (FFB) and transbronchial biopsy (TBBx) are routinely performed in lung transplant recipients to acquire clinical samples for assessing lung allograft health. TBBx is universally performed to assess and monitor allograft function when clinically indicated but is often done by many programs as surveillance in the setting of clinical stability to detect silent or subclinical allograft rejection and/or infection.^1,2^ Consensus documents provide recommendations on the histologic analysis of allograft tissue obtained by TBBx as a means for detection and grading acute cellular rejection (ACR).^3^ Currently, there are no recommendations on the timing of tissue processing, interpretation, and notification of results to transplant pulmonologists delivering direct patient care. Therefore, we completed a quality improvement initiative that spanned all 3 stages to reduce the time to diagnosis of ACR and notification of the treating transplant clinician. A rapid tissue processing system was implemented by the clinical laboratory with tissue review by the transplant pathologist immediately after completion of tissue processing and direct communication with the treating transplant pulmonologist.

## METHODS

Using an uncontrolled before and after study design as described in the medical literature,^4^ we completed a quality improvement study that included Institutional Review Board approval to obtain data from lung transplant recipients needing TBBx at our institution from September 2006 to March 2018. The rapid tissue processing system was instituted in March 2011. Data from recipients before this date served as baseline data. The rapid tissue throughput system takes about 3 hours to complete and involves formalin fixation (1 hour), tissue processing, paraffin infiltration and embedding (1 hour), block orientation and cutting (15 minutes), and staining with hematoxylin and eosin (45 minutes). After completion of tissue processing, the interpreting transplant pathologist reviews the slides and then communicates the results directly to the transplant pulmonologist by phone, which would add up to 1 more hour to the entire process.

Our surveillance FFB with TBBx are done at predetermined time intervals after lung transplant: 1, 3, 6, 9, 12, and 18 months, and then annually. We may perform additional FFB with TBBx due to clinical indications. Routine sedation for these procedures included intravenous propofol given by an anesthesiologist with topical lidocaine applied to the lower airway respiratory mucosa via the bronchoscope. The FFB with TBBx was performed with a bronchoscope and alligator biopsy forceps as determined by the age and size of the patient. A transplant pathologist performed histologic assessment of the lung allograft tissue to detect and grade ACR according to published guidelines.^3^ The primary outcome was notification of the TBBx results of the lung allograft. We compared rates of the same-day and next-day notification using chi-square or Fisher’s exact tests. Continuous variables were reported as a median and CI and compared by period using Wilcoxon rank-sum tests. Categorical variables were expressed as a count and percentage, and compared by period using Fisher’s exact tests. Data were analyzed using Stata/IC 14.2 (StataCorp, LP, College Station, TX). Two-tailed *P* < 0.05 was considered statistically significant. In addition to traditional statistics, a statistical process control chart (p-chart) was generated as a means to better assess time series analysis.

## RESULTS

**Fig. 1.**
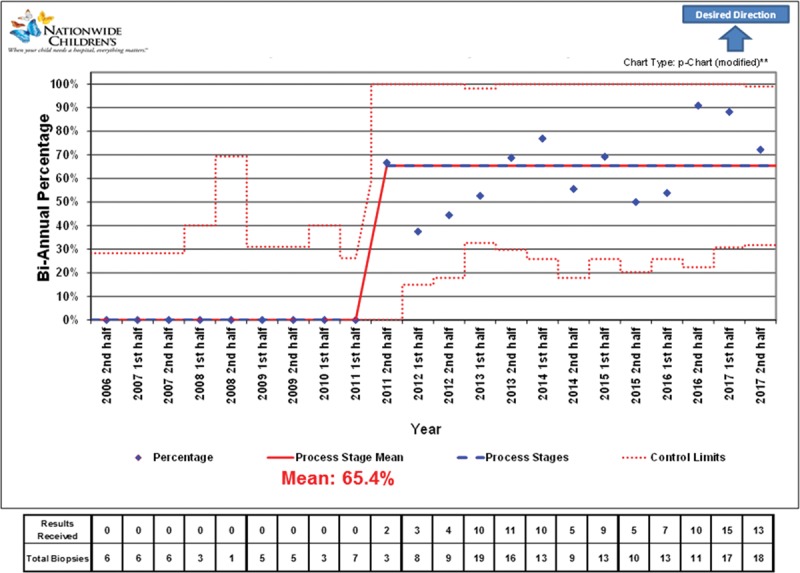
Control chart showing the percentage of patients whose results were attained the same day as transbronchial biopsies were performed. **Control limits are wider than standard because the number of 0% (or 100%) is sufficient to skew probabilities. Standard limits would yield false special cause flags.

**Fig. 2.**
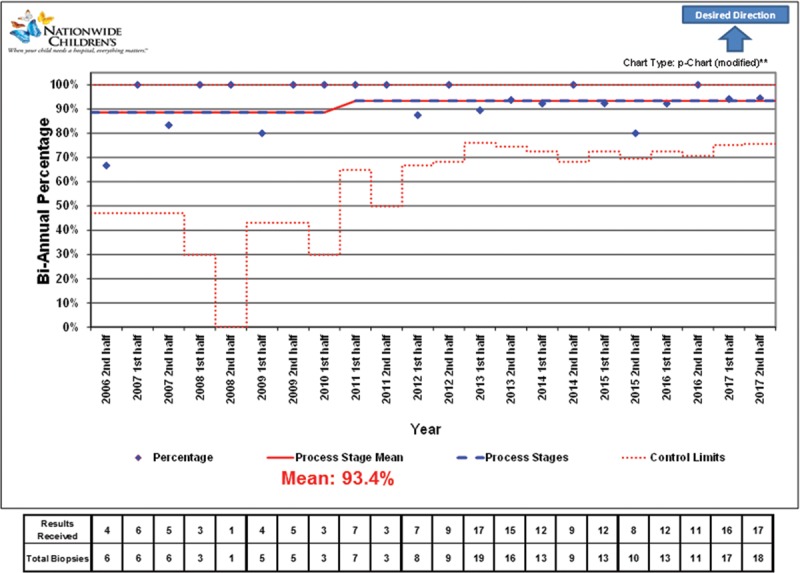
Control chart showing the percentage of patients whose results were attained by next day following transbronchial biopsies. **Control limits are wider than standard because the number of 0% (or 100%) is sufficient to skew probabilities. Standard limits would yield false special cause flags.

During the study period, 28 pediatric and young adults, 0–41 years of age at the time of lung transplant, underwent 210 TBBx (1–26 TBBx per patient). Patients had a median age of 23 years (interquartile range [IQR], 16–31). One TBBx was excluded from further analysis due to unknown notification date. Of the remaining 209 TBBx, 38 were performed before the implementation of the new diagnostic and communication system, and 171 afterward. Characteristics of TBBx before and after the change are summarized in Table 1. Figure 1 shows the percentage of patients whose results were attained the same day as TBBxs were performed after implementation of the quality improvement initiative, whereas Figure 2 shows the percentage of patients whose results were attained by next day following TBBxs.

**Table 1.**
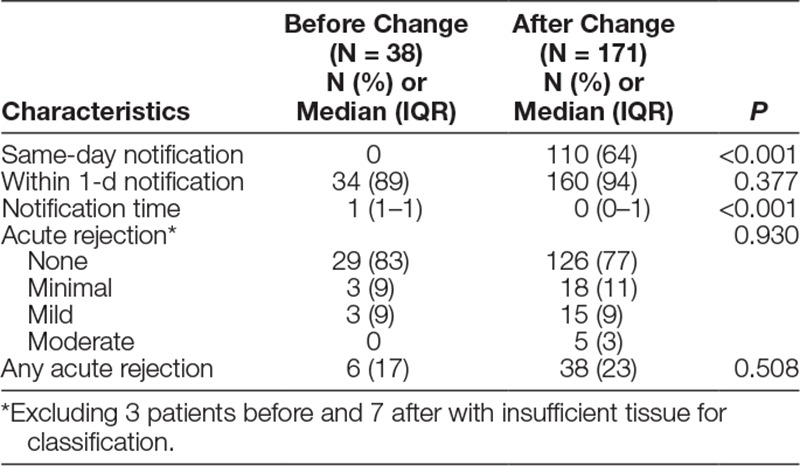
Characteristics of Transbronchial Biopsy Results According to Occurrence Before or After the Change to the Rapid Interpretation System (N = 209)

Traditional statistics identified a median time to notification following the change was 0 days (IQR, 0–1) compared with 1 day (IQR, 1–1) before the change (*P* < 0.001). Among TBBx that obtained sufficient tissue for classification (before 35, after 164), acute rejection did not differ significantly between periods, with 6 (17%) identifications of acute rejection before compared with 38 (23%) after (*P* = 0.508). After the change, same-day notification increased, with 110 (64%) TBBx resulting in same day notification compared with 0 before (*P* < 0.001). Next-day notification also increased after the change, but not significantly, with 160 (94%) TBBx resulting in the notification by the next day compared with 34 (89%) before the change (*P* = 0.377). We initiated treatment of ACR on the day of diagnosis for the entire cohort.

## DISCUSSION

As a result of this patient-centered quality improvement initiative, we minimized the time to achieving the diagnosis of ACR and initiating appropriate therapy in pediatric and young adult lung transplant recipients. The rapid throughput system required ≥3 hours for fixation, tissue processing, paraffin infiltration/embedding, block orientation/cutting, and staining, so whenever the biopsied tissue arrived in the afternoon, the diagnosis and the relevant notification of the clinical team would be delayed until the next day. However, even despite this limitation, we achieved same-day diagnosis and notification in nearly two-thirds of our patient population.

Although the rapid processing of allograft tissue was highly important in achieving the results of this project, we strongly believe that the direct and consistent communication between the interpreting transplant pathologist and the transplant pulmonologist after each TBBx was also critical in ameliorating delays in the initiation of immediate treatment of ACR. In all cases including those that were delayed until the next day, treatment for ACR was initiated the same day the diagnosis was achieved. Although there are no data on the effect of delaying ACR treatment in lung transplant recipients, we begin treating ACR as soon as possible to reduce deleterious effects and optimize long-term outcomes. Our study was not designed to determine the clinical significance of an expedited ACR diagnosis in asymptomatic or symptomatic lung transplant recipients, but there are 2 clinical scenarios where an expedited diagnosis would be highly relevant regardless: (1) the symptomatic patient with unclear reason for clinical symptoms and (2) patient with antibody-mediated rejection (AMR) with or without ACR where histopathologic features would confirm or suggest AMR as the diagnosis and support the initiation of AMR treatment, especially in the setting of a rapidly deteriorating patient.

Although this work has several limitations, including its single-center nature and small sample size, the fact that we were able to significantly reduce the time to diagnosis (and thus, to treatment) of ACR is significant because it is conceivable (although as of yet unproven) that prompt ACR treatment may improve long-term lung transplant outcomes. Our study design precluded us from determining the clinical significance of an expedited ACR diagnosis in asymptomatic or symptomatic lung transplant recipients, so this is an area that needs further research to determine the relevance of our findings. With limited improvement in post-lung transplant survival for multiple decades, patient-centered care may serve as a potential for improving these long-term outcomes. Factors that facilitated the success of this specific initiative were a commitment from key stakeholders and the multidisciplinary team, and support from an institution committed to improving quality of care of lung transplant recipients. In conclusion, the current analysis describes success with real-world experience in an area we felt needed improvement at our program. We believe that lung transplantation is an area ripe for quality improvement initiatives that could address the quality of medical care in a complex patient population.

## DISCLOSURE

The authors have no financial interest to declare in relation to the content of this article.
